# Neutrophil elastase, an acid-independent serine protease, facilitates reovirus uncoating and infection in U937 promonocyte cells

**DOI:** 10.1186/1743-422X-2-48

**Published:** 2005-05-31

**Authors:** Joseph W Golden, Leslie A Schiff

**Affiliations:** 1 Department of Microbiology, University of Minnesota, Mayo Mail Code 196, 420 Delaware St. S.E., Minneapolis, Minnesota 55455, USA

## Abstract

**Background:**

Mammalian reoviruses naturally infect their hosts through the enteric and respiratory tracts. During enteric infections, proteolysis of the reovirus outer capsid protein σ3 is mediated by pancreatic serine proteases. In contrast, the proteases critical for reovirus replication in the lung are unknown. Neutrophil elastase (NE) is an acid-independent, inflammatory serine protease predominantly expressed by neutrophils. In addition to its normal role in microbial defense, aberrant expression of NE has been implicated in the pathology of acute respiratory distress syndrome (ARDS). Because reovirus replication in rodent lungs causes ARDS-like symptoms and induces an infiltration of neutrophils, we investigated the capacity of NE to promote reovirus virion uncoating.

**Results:**

The human promonocyte cell line U937 expresses NE. Treatment of U937 cells with the broad-spectrum cysteine-protease inhibitor E64 [*trans*-epoxysuccinyl-L-leucylamido-(4-guanidino)butane] and with agents that increase vesicular pH did not inhibit reovirus replication. Even when these inhibitors were used in combination, reovirus replicated to significant yields, indicating that an acid-independent non-cysteine protease was capable of mediating reovirus uncoating in U937 cell cultures. To identify the protease(s) responsible, U937 cells were treated with phorbol 12-myristate 13-acetate (PMA), an agent that induces cellular differentiation and results in decreased expression of acid-independent serine proteases, including NE and cathepsin (Cat) G. In the presence of E64, reovirus did not replicate efficiently in PMA-treated cells. To directly assess the role of NE in reovirus infection of U937 cells, we examined viral growth in the presence of N-Ala-Ala-Pro-Val chloromethylketone, a NE-specific inhibitor. Reovirus replication in the presence of E64 was significantly reduced by treatment of cells with the NE inhibitor. Incubation of virions with purified NE resulted in the generation of infectious subviron particles that did not require additional intracellular proteolysis.

**Conclusion:**

Our findings reveal that NE can facilitate reovirus infection. The fact that it does so in the presence of agents that raise vesicular pH supports a model in which the requirement for acidic pH during infection reflects the conditions required for optimal protease activity. The capacity of reovirus to exploit NE may impact viral replication in the lung and other tissues during natural infections.

## Background

Mammalian reoviruses are the prototypic members of the *Reoviridae *family, which also includes the pathogenic rotaviruses, coltiviruses, seadornaviruses and orbiviruses. These viruses share elements of their replication cycle as well as structural features, including a non-enveloped multi-layered capsid that surrounds a segmented dsRNA genome. In humans, mammalian reoviruses are typically associated with mild and self-limiting enteric and respiratory infections. However, studies in neonatal mice reveal that reoviruses can spread to distant tissue sites in immunocompromised hosts (reviewed in[1]). The factors that determine reovirus cellular host range are poorly understood. Because reovirus attaches to cells through interactions with broadly expressed receptors, one or more subsequent steps in the viral life cycle must help to regulate host range and pathogenesis. Our recent studies suggest that one such step is proteolysis of the capsid protein σ3 [2,3].

In cell culture, the first step in infection is attachment to cellular receptors through interactions with the viral protein σ1 [4,5]. σ1 interacts with two known receptors: sialic acid and junctional adhesion molecule 1 [6-8]. Following binding, virions are internalized by receptor-mediated endocytosis [9]. Endocytosis is an essential step in the viral life cycle under standard infection conditions [10]. Within the endosomal and/or lysosomal compartment, proteases convert virions into particles that resemble *in vitro*-generated intermediate subvirion particles (ISVPs) [10-14]. These uncoating intermediates, typically prepared using chymotrypsin or trypsin, lack σ3 and have a cleaved form of μ1. Studies using ISVPs and ISVPs recoated with recombinant outer capsid proteins reveal that σ3 plays a key role in regulating reovirus cell entry by interacting with, protecting, and controlling the conformational status of the underlying penetration protein μ1 [15-18]. In cells that cannot efficiently mediate σ3 degradation during uncoating, reovirus infection is slow or blocked; these cells can be productively infected by particles that lack σ3 [2]. *In vitro*, ISVP-like particles can be generated by a variety of proteases in addition to chymotrypsin and trypsin, including proteinase K, thermolysin, endoproteinase lys-C, Cat L, Cat B and Cat S[3,19-21].

Recent work has provided insight into the cellular determinants of reovirus uncoating. In murine fibroblasts, where reovirus entry has been best studied, the cysteine proteases Cat L, and to a lesser extent Cat B, are required for σ3 removal, whereas the aspartyl protease Cat D is not [14,21-25]. Virion disassembly in murine fibroblasts also requires acidic pH[10,26,27]. Recently, we demonstrated that reovirus uncoating in the macrophage-like cell line P388D is mediated by the acid-independent lysosomal cysteine protease Cat S[3]. This finding revealed that in different cell types, distinct proteases can facilitate reovirus uncoating. Our results suggested a model in which infection in some cells is acid-dependent because the proteases that mediate σ3 removal in those cells require acidic pH for maximal activity. Thus, in fibroblasts or other cells in which the acid-dependent proteases Cat L and Cat B mediate σ3 removal, infection is acid-dependent [21,23,28], whereas in Cat S-expressing cells it is not [3], because Cat S maintains its activity at neutral pH [29]. Insight from the analysis of reovirus cell entry facilitated the recent discovery that activation of the Ebola virus glycoprotein also depends on the activity of the acid-dependent endosomal proteases Cat B and Cat L [30].

The role that specific intracellular and extracellular proteases play in regulating reovirus tropism, spread, and disease in animals is largely unknown, except in the murine intestinal tract where pancreatic serine proteases have been shown to mediate σ3 removal [31,32]. Reovirus also naturally infects hosts via the respiratory tract [33-35]. One protease with well-described effects in the respiratory tract is elastase 2 (GenBank NM_001972), an inflammatory serine protease of the chymotrypsin family, which is predominantly expressed by neutrophils [36]. NE plays a prominent role in wound repair [37-39] and in controlling microbial infections [38-40]. NE expression can also promote pathogenesis; it has been implicated in smoke-induced emphysema [41], respiratory syncytial viral bronchiolitis [42] and in the respiratory syndrome ARDS [4]. The fact that reovirus replication in the rodent lung causes an influx of neutrophils [35,43] and that reovirus infection can recapitulate ARDS [44], led us to ask whether NE could mediate productive reovirus uncoating. We investigated reovirus infection in the monocyte-like cell line U937, because it is known to express NE [45]. Experiments described in this report demonstrate that reovirus infection in U937 cells does not require cysteine protease activity and is not blocked in the presence of agents that raise vesicular pH. Studies using protease inhibitors suggest that, in the absence of cysteine protease activity, NE is largely responsible for productive infection of U937 cells. NE can directly mediate σ3 removal from reovirus virions; the resultant particles are infectious and do not require additional intracellular proteolysis. Our data raise the possibility that NE is involved in reovirus replication in the respiratory tract.

## Results

### Reovirus infection of U937 cells does not require cysteine protease activity

**Figure 1:**
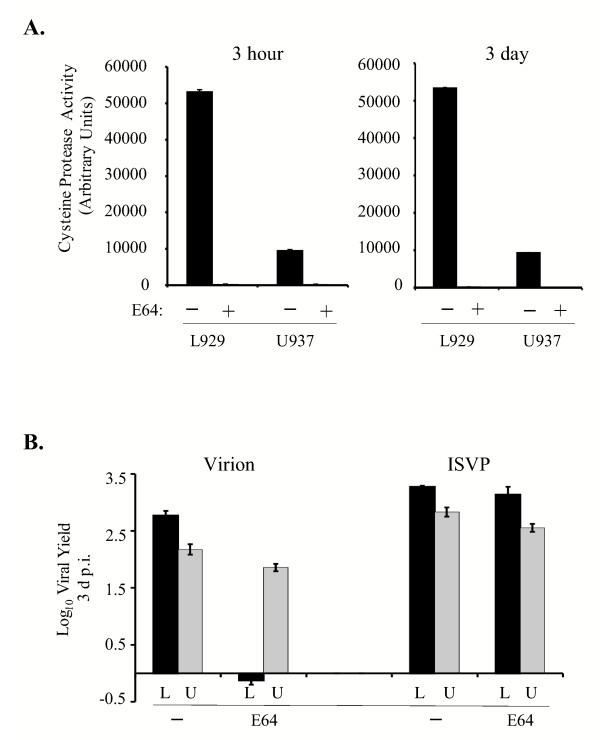
**Analysis of viral replication in L929 and U937 cells treated with E64. **A. 3 × 10^6 ^L929 and U937 cells were untreated (-; black) or treated (+; grey) with 300 μM E64 for 3 h or 3 d. Cysteine protease activity was assessed using the fluorogenic substrate Z-Phe-Arg-MCA (Sigma) and plotted in arbitrary units. Activity levels in treated cells were so low (in L939 cells, 254 units at 3 h and 231 units at 3 d; in U937 cells, 200 units at 3 h and 115 units at 3 days) that they cannot be visualized on this graph. B. L929 (L; black bars) and U937 (U; grey bars) cells were treated with 300 μM E64 for 3 h prior to infection. Cells were then infected with reovirus strain Lang virions or ISVPs at an MOI of 3. Infectious virus present at 3 d p.i. was determined by plaque assay on L929 cell monolayers. Each time point represents the mean (+/- SD) derived from three independent samples.

The promonocytic cell line U937 expresses large amounts of elastase [45] and provided a suitable system to analyze the role of this protease in reovirus infection. To determine if NE can facilitate reovirus infection of U973 cells, we first established conditions under which lysosomal cysteine protease activity was inhibited. Cells were treated with 300 μM E64, a broad-spectrum cysteine protease inhibitor [46], and protease activity was assessed using the Cat L and Cat B-specific fluorogenic substrate Z-Phe-Arg-MCA. We analyzed enzyme activity at two time points: first after 3 h of treatment, because we typically pre-treat cells with inhibitors for 3 h prior to infection, and second at 3 d, the time point at which viral yield would be quantified. As shown in Fig. 1A, treatment with 300 μM E64 completely abolished cysteine protease activity in U937 cells. Consistent with our previous findings [3], E64 also completely blocked cysteine protease activity in L929 cells. Raw values are provided, to illustrate the relative difference in Cat L/B enzyme activity levels between U937 cells and L929 fibroblasts. In the absence of inhibitor, Cat L and B activity was significantly lower in U937 cells than in L929 cells. This may be a consequence of high expression in U937 cells of cystatin F, an intracellular cysteine protease inhibitor with specificity for Cat L and papain [47].

Next, we compared reovirus replication in E64-treated U937 and L929 cells. Cells were pre-treated for 3 h and infected with Lang virions or ISVPs at a multiplicity of infection (MOI) of 3. The results of a representative experiment are shown in Fig. 1B. In the absence of E64, both L929 and U937 cells supported reovirus replication, consistent with the fact that these cells express Cat L. As expected, E64 blocked virion infection of L929 cells; however, viral yields in E64-treated U937 cells were only slightly reduced relative to untreated cells. ISVPs, which lack capsid protein σ3, replicated efficiently in treated cells, indicating that 300 μM E64 was not toxic to either cell type. These results demonstrate that productive infection of U937 cells by Lang virions does not require the activity of E64-sensitive, papain-like cysteine proteases.

### Infection of U937 cells is acid-independent

**Figure 2:**
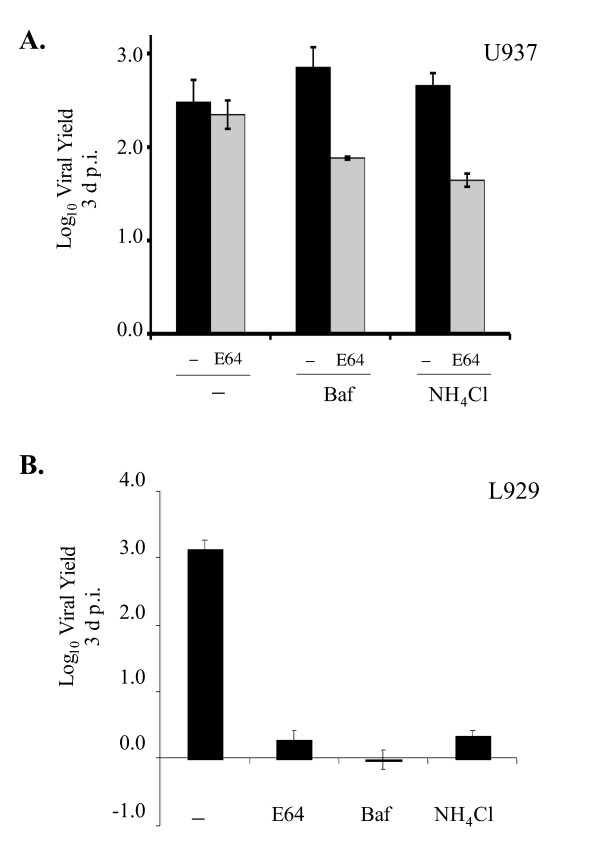
**Effects of agents that raise vesicular pH on reovirus replication in U937 and L929 cells. **A. U937 cells were pre-treated without (-; black bars) or with 300 μM E64 (E64; grey bars) in the presence or absence of 25 nM Baf or 20 mM NH_4_Cl. Following pre-treatment, cells were infected with reovirus strain Lang at an MOI of 3 and viral yield was measured at 3 d.p.i as described in the legend to Fig. 1B. B. L929 cells were pre-treated without (-) or with E64, 25 nM Baf or 20 mM NH_4_Cl. Pre-treated cells were infected with reovirus strain Lang at an MOI of 3 and viral yield was measured at 3 d p.i. as described in the legend to Fig. 1B.

Acidic pH is required for productive reovirus infection of murine L929 fibroblasts [10,27], in which the acid-dependent proteases Cat L and Cat B mediate uncoating [21,23]. Serine proteases, including NE, and metalloproteases function over a broader pH range. Therefore, to gain insight into the nature of the protease(s) that can promote reovirus uncoating in U937 cells, we investigated the requirements for acidic pH. L929 and U937 cells were left untreated or pre-treated with E64 in the presence or absence of bafilomycin A1 (Baf) or NH_4_Cl. These latter agents raise vesicular pH by blocking the vacuolar H^+^-ATPase pump or by acting as a weak base, respectively [48-50]. After pre-treatment, cells were infected with Lang virions at an MOI of 3 and viral yields were determined at 3 days post infection (d p.i.). A representative experiment is shown in Fig. 2. Treatment with either Baf or NH_4_Cl did not inhibit viral replication in U937 cells; yields reached 2.9 and 2.7 logs, respectively. Furthermore, these agents had little effect on viral replication in U937 cells even when the cells were also treated with E64 to inhibit cysteine protease activity. In contrast, Baf or NH_4_Cl alone completely blocked reovirus replication in L929 cells, consistent with the requirement for Cat L/B-mediated σ3 removal in these cells. Given that reovirus uncoating is an essential step in the viral life cycle [10], these findings revealed that a non-cysteine protease that functions at neutral pH can facilitate this step in U937 cells.

### Analysis of reovirus replication in U937 cells differentiated by PMA

**Figure 3:**
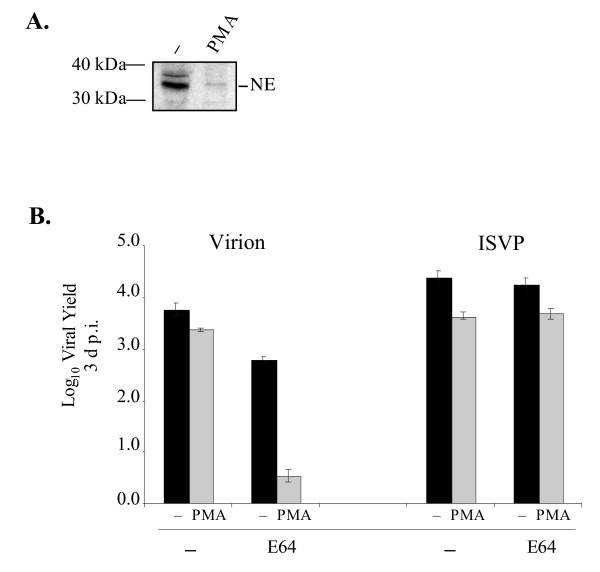
**Analysis of reovirus replication in U937 cells differentiated with PMA. **A. Lysates generated from 10^5 ^U937 cells that were untreated (-) or treated with 150 nM PMA for 72 h were resolved on SDS-12% polyacrylamide gels and electrophoretically transferred to a nitrocellulose filter. The filter was subsequently incubated with a polyclonal goat antibody against human NE (1:400) (Santa Cruz Biotechnology). The filter was washed and incubated with a secondary anti-goat antibody conjugated to horseradish peroxidase (1:5000) (Santa Cruz Biotechnology). Protein bands were detected using reagents that generate a chemiluminescent signal (Amersham). B. U937 cells that were undifferentiated (-; black bars) or differentiated (PMA; grey bars) with 150 nM of PMA for 72 h were left untreated (-) or were treated with 300 μM E64. Following pre-treatment with the protease inhibitor, cells were infected with Lang virions or ISVPs at an MOI of 3. Viral yield was quantified at 3 d p.i. as described in the legend to Fig. 1B.

Treatment of the promonocytic U937 cells with phorbol ester derivatives results in their differentiation into macrophage-like cells [51,52]. This differentiation is characterized by several major phenotypic changes, including increases in expression of urokinase plasminogen activator receptors, upregulation of collagenase activity and a significant decrease in the expression of NE and Cat G [51,52]. We predicted, therefore, that PMA treatment might decrease the capacity of reovirus virions to replicate in U937 cells when cysteine proteases were inhibited. To confirm that there was a significant decrease in NE expression in U937 cells differentiated with PMA, U937 cells were treated with 150 nM PMA for 72 h and expression of NE was analyzed by immunoblotting. As shown in Fig. 3A, NE was expressed in untreated U937 cells, but its expression was dramatically reduced following PMA-induced differentiation.

To examine the effect of U937 cell differentiation on reovirus infection, PMA-treated and untreated U937 cells were left untreated or were treated with E64 for 3 h and infected with Lang virions or ISVPs at an MOI of 3. Yields were measured at 3 d p.i. and the results of a typical experiment are shown in Fig. 3B. In the absence of E64, PMA-treated U937 cells were permissive to infection by virions. PMA treatment only decreased yields by ~0.5 log relative to untreated cells. In contrast, when PMA-differentiated U937 cells were treated with E64 to inhibit cysteine protease activity, they no longer supported productive infection by Lang virions. Because these results could be explained if E64 was toxic to PMA-treated U937 cells, we examined the replication of ISVPs. In the presence of E64, ISVPs replicated to high yields in both undifferentiated and differentiated U937 cells. Since PMA-induced differentiation of U937 cells caused a substantial decrease in NE expression, these results are consistent with the hypothesis that NE or another similarly regulated neutral protease facilitates productive reovirus infection in promonocytic (pre-differentiated) U937 cells.

### NE can facilitate reovirus infection in U937 cells

We directly examined the capacity of NE to facilitate reovirus infection by using the irreversible elastase inhibitor, N-(methoxysuccinyl)-Ala-Ala-Pro-Val-chloromethyl ketone [53]. This inhibitor is highly specific for NE and does not inhibit the activity of the related serine protease, Cat G [53]. First, we established the efficacy and specificity of inhibitor treatment under our experimental conditions. U937 cells were treated with the NE inhibitor, E64, Baf or NH_4_Cl for either 3 h or 2 d and the activity of NE in cell lysates was examined using a colorimetric substrate. As shown in Table 1, the NE inhibitor was active at both time points. In cells treated with the specific inhibitor, NE activity was less than 9% of that in untreated U937 cells. In contrast, in U937 cells treated with E64, Baf or NH_4_Cl, NE activity was only modestly reduced, remaining above 80% even after 2 d. These results are consistent with the capacity of NE to function at neutral pH. To verify the specificity of the NE inhibitor, we also examined its effect on Cat L/B activity using the fluorogenic substrate Z-Phe-Arg-MCA. As expected, Cat L/B activity was completely inhibited by E64 but largely unaffected by the NE inhibitor.

**Table 1:** Inhibition of NE activity in U937 cells.^a^

	% NE Activity^b^				% Cat Activity^c^	
	
Time post-treatment	NE inhibitor	E64	Baf	NH_4_Cl	E64	NE inhibitor
3 h	7.5 ± 5.0	84.3 ± 5.8	87.0 ± 9.0	88.3 ± 9.4	ND	ND
2 d	8.8 ± 3.5	89.5 ± 9.1	88.8 ± 10.0	86.3 ± 3.6	-4.7 ± 1.6	98.8 ± 3.9

^a^U937 cells were treated with the indicated inhibitors for 3 h or 2 d.

^b^NE activity was assessed using the colorimetric substrate MeOSuc-Ala-Ala-Pro-Val-ρNA and percent activity relative to untreated cells was calculated.

^c ^Cathepsin L and B activity were assessed using the fluorogenic substrate Z-Phe-Arg-MCA and percent activity relative to untreated cells was calculated.

**Figure 4:**
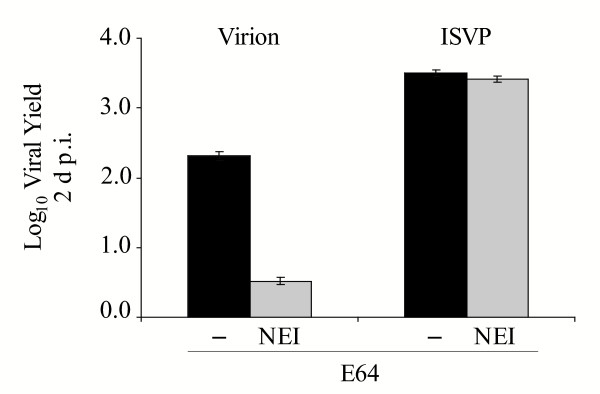
**Analysis of viral replication in U937 cells treated with E64 and NE inhibitor. **U937 cells were pre-treated with 300 μM E64 in the absence (-; black bars) or presence (NEI; grey bars) of 200 μM N-Ala-Ala-Pro-Val chloromethylketone, an inhibitor of NE. Following inhibitor pre-treatment, cells were infected with Lang virions or ISVPs at an MOI of 3. Viral yield was analyzed at 2 d p.i. as described in the legend to Fig. 1B.

To examine the effect of the NE inhibitor on reovirus replication in U937 cells, we pre-treated them for 3 h with E64 in the presence or absence of the NE inhibitor, infected them with Lang virions or ISVPs at an MOI of 3, and quantified viral yields at 2 d p.i. A representative experiment is shown in Fig. 4. Consistent with the results shown in Fig 1, virion replication was not blocked in E64-treated U937 cells. However, in the presence of both E64 and the NE inhibitor, yields were significantly reduced. ISVPs replicated to high yields in treated cells, indicating that the combination of inhibitors was not toxic to U937 cells. These results demonstrate that NE plays a critical role in reovirus infection of U937 cells when cysteine proteases are inhibited.

### NE-generated subviral particles are infectious and do not require additional proteolytic processing

**Figure 5:**
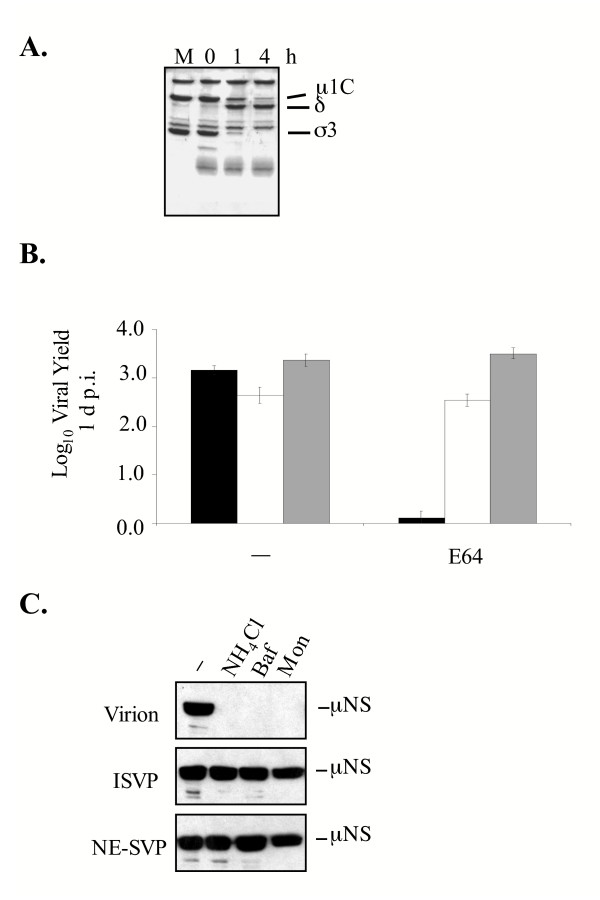
**Analysis of NE treatment of reovirus virions. **A. Lang virions (7.5 × 10^10^) were incubated with purified 25 μg/ml NE (Calbiochem) at 37°C for the indicated times (in min) and analyzed on SDS-12% polyacrylamide gels and proteins were visualized by Coomasssie staining. The mock sample (M) consisted of virions held in reaction buffer in the absence of protease for 4 h. The positions of reovirus capsid proteins are labeled. B. L929 cells were left untreated (-) or were pre-treated with 300 μM E64 and infected with Lang virions (black bars), ISVPs (white bars) or NE-treated particles (grey bars) at an MOI of 3. Viral yield was determined at 1 d p.i. C. L929 cells were left untreated (-) or were pre-treated with 20 mM NH_4_Cl, 25 nM Baf or 25 μM monensin and infected with Lang virions or protease-treated particles. Cell extracts prepared at 1 d p.i. were analyzed for the expression of the viral non-structural protein μNS by immunoblotting using antiserum against μNS (diluted 1:12500).

NE, like many cellular proteases, is expressed as a proenzyme that becomes activated only after its pro-region is removed [54]. We envisioned two models by which NE could facilitate reovirus infection of U937 cells. In the first, NE could directly mediate σ3 degradation, leading to the generation of an ISVP-like particle. In the second, NE could act indirectly by activating another protease. To try to distinguish between these models, we examined the capacity of purified NE to directly mediate σ3 removal from Lang virions *in vitro*. Purified Lang virions were treated with NE for 1 and 4 h and the treated virus particles were analyzed by SDS-PAGE. As shown in Fig. 5A, NE efficiently removed σ3 from Lang virions; after 1 h very little intact σ3 remained on viral particles. After 4 h of NE treatment, σ3 was completely removed and the underlying μ1C was cleaved to the δ and φ fragments (φ was not retained on the gel). When we assayed the infectivity of the resultant particles by plaque assay we found that NE treatment did not negatively affect the titer of Lang particles (data not shown).

To determine if NE-generated SVPs required further proteolytic processing of σ3, L929 cells were pre-treated with E64 to block cysteine protease activity and infected at an MOI of 3 with Lang virions, ISVPs or NE-generated subviral particles (NE-SVPs). Viral yields were determined at 1 d p.i. As expected, E64 blocked infection of L929 cells by virions. In contrast, both ISVPs and NE-SVPs replicated efficiently in the presence of the cysteine protease inhibitor (Fig. 5B). Because virion disassembly in L929 cells requires acidic pH [10], we also examined the capacity of NE-SVPs to infect L929 cells treated with Baf, NH_4_Cl or monensin, three agents that raise vesicular pH by distinct mechanisms. Cells were treated with these agents and then infected with virions, ISVPs or NE-SVPs at an MOI of 10. At 18 hours post infection (h p.i.), cell lysates were harvested and expression of the reovirus non-structural protein μNS was analyzed by immunoblotting (Fig. 5C). As expected, when treated cells were infected with virions, viral protein expression was blocked. In contrast, μNS expression was evident even in the presence of agents that raise pH when infections were initiated with ISVPs or NE-SVPs (Fig. 5C). Together, these results demonstrate that NE can directly mediate σ3 removal from virions to generate infectious particles that do not require further proteolytic processing by acid-dependent cysteine proteases in L929 cells.

## Discussion

### NE, an acid-independent serine protease, can promote productive reovirus infection in U937 promonocytes

Serine proteases are involved in reovirus infection in the mammalian intestinal tract [31] and in this report we provide evidence that they can mediate uncoating and promote infection in U937 cells. This expands the range of proteases that promote reovirus infection in cell culture to include NE as well as the cysteine proteases Cat L, Cat B, and Cat S. Several lines of evidence now support the notion that protease expression is a cell-specific host factor that can impact reovirus infection. For example, some reovirus strains are inefficiently uncoated by Cat S and thus do not replicate to high yield in P388D macrophages [3]. In this report we demonstrate that PMA-induced differentiation influences the type of protease that mediates reovirus uncoating in U937 cells. In these cells, PMA treatment is reported to increase Cat L expression [55] and decrease expression of the serine proteases NE and Cat G [56,57]. Accordingly, when we used PMA to induce U937 cell cultures to differentiate, reovirus infection became sensitive to the cysteine protease inhibitor E64. We suspect that Cat L is largely responsible for uncoating in these PMA-differentiated cells, but the acid-independent protease Cat S may also play a role. We are currently addressing this question by analyzing infection in PMA-differentiated cells treated with either Baf or NH_4_Cl.

### Does the serine protease Cat G also play a role in reovirus infection of U937 cells?

Our data do not completely resolve this question. Cat G is expressed by U937 cells and, like NE, it is down-regulated by PMA treatment. Furthermore, we found that *in vitro *treatment of reovirus virions with purified Cat G generates SVPs that behave like NE-SVPs in that they are infectious in the absence of further proteolytic processing (data not shown). Results of our experiment with the NE-specific inhibitor suggest that NE is largely responsible for the E64-resistant infection in U937 cells. While this inhibitor is reported not to inhibit Cat G [53], we have not independently confirmed this. Another approach to assess the role of Cat G in reovirus infection of U937 cells would be to examine the effect of Cat G-specific inhibitors on infection. We tried one such inhibitor, Cathepsin G Inhibitor I (Calbiochem)[58], but found that it was cytotoxic to U937 cell cultures. Given that both NE and Cat G can generate infectious reovirus SVPs, more work needs to be done in order to understand the role that these two proteases play in infection in these cells.

### The acid-dependence of reovirus infection is a reflection of the requirements for protease activation

Previously, we reported that virion uncoating mediated by Cat S does not require acidic pH [3]. These results were consistent with the acid-independence of Cat S activity [37]. Together, the results in Fig. 2 and Fig. 4 reveal that, like Cat S, NE-mediates infection in an acid-independent manner. This finding thus provides further support for a model in which the requirement for acidic pH during reovirus infection of some cell types reflects the requirement for acid-dependent protease activity in those cells rather than some other requisite acid-dependent aspect of cell entry. The small effect of Baf and NH_4_Cl on E64-resistant reovirus growth (Fig. 2) may reflect the participation of one or more acid-dependent proteases (such as Cat D) in the activation of NE.

### Does NE mediate uncoating intracellularly or extracellularly in U937 cell cultures?

Elastase is stored in azurophilic granules that are the major source of acid-dependent hydrolases in neutrophils [59]. Although these granules do not contain LAMP-1 or LAMP-2 [60] they contain the lysosomal markers LAMP-3 [61] and CD68 [62] and are accessible to endocytosed fluid-phase markers under conditions of cellular stimulation [63]. NE can be released from neutrophils during degranulation [64] and its cell surface expression can be induced upon PMA treatment [65]. However, studies in U937 cells have shown that NE is predominantly retained intracellularly and that little if any activity is present in the extracellular medium [45]. Consistent with this, we have been unable to generate ISVP-like particles by treatment of virions with U937 culture supernatants (data not shown). This observation, together with our finding that PMA treatment decreases the capacity of E64-treated U937 cells to support reovirus infection, leads us to favor a model in which NE-mediated virion uncoating in U937 cell cultures occurs intracellularly.

### Implications for infection in the host

*In vivo*, a number of viruses, including dengue and respiratory syncytial virus, induce the release of IL-8, a cytokine that serves as a chemoattractant for neutrophils and promotes their degranulation [66,67]. Reovirus replication in the rat lung results in neutrophilic invasion [35,43] and studies in cell culture indicate that reovirus infection can induce IL-8 expression [68]. Thus, the capacity of reovirus to induce IL-8 secretion *in vivo *might facilitate the release of neutrophilic lysosomal hydrolases, including NE, into the extracellular milieu. In this report, we have shown that mammalian reovirus can utilize this acid-independent serine protease for uncoating. Our data suggest that, *in vivo*, one consequence of reovirus-induced IL-8 expression would be the generation of infectious NE-SVPs. Like ISVPs, these particles would be predicted to have an expanded cellular host range because they can infect cells that restrict intracellular uncoating [2]. Thus, inflammation might be predicted to exacerbate reovirus infection by promoting viral spread. Future studies using mice with deletions in the NE gene will be required to elucidate the role this protease plays during reovirus infection in the respiratory tract and other tissues. Finally, given the recent finding that endosomal proteolysis of the Ebola virus glycoprotein is necessary for infection [30], our results raise the interesting possibility that NE or other neutrophil proteases may play a role in cell entry of other viruses.

## Methods

### Cells and viruses

Murine L929 cells were maintained as suspension cultures as described previously [Kedl, 1995 #94]. U937 cells were maintained in RPMI medium (GIBCO-BRL, Gaithersburg, MD) supplemented to contain 10% fetal calf serum (Gibco-BRL), 50-units/ml penicillin (GIBCO-BRL), 50 μg/ml streptomycin (GIBCO-BRL) and 2 mM glutamine (GIBCO-BRL). Where indicated, U937 cells were differentiated by treatment with 150 nM of PMA (Sigma) for 48 h prior to infection.

Third-passage lysate stocks of reovirus were prepared in L929 cell cultures. Purified virions were prepared by CsCl density gradient centrifugation of extracts from cells infected with third-passage lysate stocks [Furlong, 1988 #81]. ISVPs were prepared by treating purified virions with chymotrypsin as described elsewhere [Nibert, 1992 #95].

### Measurement of cysteine protease activity

Cysteine protease activity was measured as described previously [23] with some minor modifications. Briefly, P388D U937 and L929 cells (2 × 10^6 ^each) were incubated in the presence or absence of 300 μM E-64, 5 nM LHVS, or 5 μM CA074 for the times indicated. After incubation, cells were trypsinized, collected by centrifugation at 179 × *g *for 10 min at 4°C and washed once in PBS. Cell pellets were resuspended in 100 μl of lysis buffer (100 mM sodium acetate [pH 5.5], 1 mM EDTA, and 0.5% Triton X-100), incubated on ice for 30 min and cell debris was pelleted by centrifugation at 89 × *g *for 10 min at 4°C. For each sample, 20 μl of clarified cell lysate was added to 80 μl of reaction buffer (100 mM sodium acetate [pH 5.5], 1 mM EDTA, 4 mM dithiothreitol) in a well of a black 96-well plate (Corning). To measure Cat B activity, 100 μM Z-Arg-Arg-7-amido-4-methylcoumarin (Z-Arg-Arg MCA) (Calbiochem) was included in the reaction buffer. To measure Cat L and Cat B activity, 100 μM Z-Phe-Arg-7-amido-4-methylcoumarin (Z-Phe-Arg-MCA) (Calbiochem) was added to the reaction buffer. Reactions were incubated for 30 min at room temperature with gentle tapping every 10 min. Fluorescence was measured using an FL600 microplate reader (Bio-Tek Instruments, Inc., Winooski, VT) with an excitation of 390 nm and emission at 460 nm.

### Measurement of serine protease activity

NE activity was determined by incubating 3 × 10^6 ^U937 cells in the presence or absence of 200 μM NE inhibitor (N-(methoxysuccinyl)-Ala-Ala-Pro-Val-chloromethyl ketone) (Sigma) for the indicated times. After treatment, cells were collected by centrifugation at 179 × g for 10 min at 4°C, washed twice in PBS and lysed in TLB (10 mM Tris [pH 7.5], 2.5 mM MgCλ_2_, 100 NaCl, 0.5% Triton X-100, 5 μg/μl of leupeptin [Sigma], 1 mM PMSF) for 30 m on ice. The lysate was clarified by centrifugation at 89 × *g *for 10 min at 4°C. For each sample, 20 μl of cell lysate was added to 80 μl of virion dialysis buffer (VDB) (150 mM NaCl, 10 mM MgCl_2_, 10 mM Tris [pH 7.5]) containing 500 μM of NE substrate (MeOSuc-Ala-Ala-Pro-Val-pNA) (Calbiochem) and incubated for 30 min at room temperature with gentle tapping every 10 min. Absorbance was measured at 405 nm using an EL340 BioTek microplate reader (Bio-Tek Instruments).

### Analysis of viral growth

Cells were infected (in triplicate) at the indicated MOI and adsorption was allowed to proceed for 1 h on ice at 4°C. After adsorption, cells were pelleted by low speed centrifugation and resuspended in fresh media. Virus and cells were then added to dram vials (2 × 10^5 ^cells/vial) containing 1 ml of chilled medium. Prior to infection, some cells were pre-treated for 3 h with 300 μM E64 and/or 25 nM Baf (Sigma), 20 mM NH_4_Cl (Sigma) and 200 μM NE inhibitor. Inhibitors were included in the medium throughout the time course for treated samples. Time zero samples were immediately frozen at -20°C and remaining samples were incubated at 37°C until the desired time point was reached. Samples were frozen and thawed three times and titrated by plaque assay on L929 cells as described elsewhere [69]. Viral yields were calculated according to the following formula: log_10_(PFU/ml)_t = x hr _- log_10 _(PFU/ml)_t = 0 _+/- standard deviation (SD).

### *In vitro *analysis of NE-mediated uncoating

NE digestions were performed as follows. Purified virions (7.5 × 10^10^) were incubated with 25 μg/ml of purified NE (Calbiochem) in 20 μL VDB at 37°C for the times indicated. Mock-treated samples were incubated in VDB for the longest time point. 1 mM PMSF and 200 μM of NE inhibitor were added to the samples to terminate the reactions. Protein sample buffer (0.125 M Tris [pH 8.0], 1% SDS, 0.01% bromphenol blue, 10% sucrose, and 5% β-mercaptoethanol) was added to each reaction mixture and samples were resolved on SDS-12% polyacrylamide gels. The protein gels were stained with Coomassie Brilliant Blue.

### Immunoblot analysis of NE expression

To analyze NE expression, cell lysates were generated from U937 cells, either treated or untreated for 48 h with 150 nM PMA as described for the analysis of viral protein expression. Lysate from the equivalent of 1 × 10^6 ^cells was run on SDS-12% polyacrylamide gels and transferred to nitrocellulose. Membranes were blocked overnight in TBST containing 10% nonfat dry milk. NE expression was analyzed using a polyclonal antibody against NE (1:400 in TBST) (Santa Cruz Biotechnology Inc, Santa Cruz, CA). Membranes were washed with TBST and incubated with a horseradish peroxidase-conjugated anti-goat IgG (1:5000 in TBST). Bound antibody was detected by treating the nitrocellulose filters with enhanced chemiluminescence (ECL) detection reagents (Amersham) and exposing them to Full Speed Blue X-ray film (Henry Schein, Melville, NY).

### Analysis of viral protein expression in infected cells

Cells were plated at 10^6^/well in a 6-well plate 18–24 h prior to infection. Virus was allowed to adsorb to cells for 1.5 h at 4°C. At this temperature, virus binds to cells but is not internalized [70]. After adsorption, the cultures were incubated at 37°C in fresh medium. Prior to some infections, cells were pre-treated for 3 h with 300 μM E64, 100 nM Baf, 25 μM monensin (Sigma), or 20 mM NH_4_Cl. In those instances inhibitors were also included in the post-adsorption culture medium. At the indicated times p.i., cells were collected by centrifugation at 179 × g, washed twice in chilled PBS and lysed in TLB. After centrifugation at 179 × g to remove cellular debris, samples were resuspended in sample buffer. Protein samples (representing 1 × 10^5 ^cells) were analyzed by electrophoresis on SDS-12% polyacrylamide gels and transferred to nitrocellulose membranes for 2 h at 100 V in 25 mM Tris-192 mM glycine-20% methanol. Nitrocellulose membranes (Bio-Rad Laboratories, Hercules, Calif.) were blocked overnight at 4°C in TBST (10 mM Tris [pH 8.0], 150 mM NaCl and 0.05% Tween) containing 5% nonfat dry milk, rinsed with TBST, and incubated with a rabbit anti-μNS polyclonal antiserum [71] (1:12500 in TBST) for 1 h. Membranes were subsequently washed with TBST and incubated for 1 h with horseradish peroxidase-conjugated anti-rabbit immunogloblin G (IgG) (1:7500 in TBST) (Amersham, Arlington Heights, Ill.). Bound antibody was detected by treating the nitrocellulose filters with enhanced chemilumescence (ECL) detection reagents (Amersham) and exposing the filters to Full Speed Blue X-ray film (Eastman Kodak, Rochester, N.Y.).

### Generation of NE subviral particles for infection

Purified virions (1.4 × 10^11^) were incubated with 25 μg/ml of purified neutrophil elastase (Calbiochem) in 40 μL of VDB at 37°C for 3 h. Reactions were terminated by adding 1 mM PMSF and 200 μM NE inhibitor to the reaction mixture. 5.0 × 10^10 ^particles were run on SDS-12% polyacrylamide gels stained with Coomassie Brilliant Blue to confirm the removal of σ3. Viral infectivity was determined by plaque assay on L929 cell monlayers.

### Analysis of virus titer after NE treatment of virions

Purified Lang virions (1.4 × 10^11^) were treated with 25 μg/ml of NE in 40 μL of VDB at 37°C for the times indicated. Reactions were terminated as described above. To verify σ3 removal, the proteins from 5.0 × 10^10 ^particles were separated on SDS-12% polyacrylamide gels and visualized with Coomassie Brilliant Blue staining. Viral infectivity for each time point was determined by plaque assay on L929 cell monolayers.

## Competing interests

The author(s) declare that they have no competing interests.

## Authors' contributions

J.W.G. performed all experiments and was responsible for the experimental design and data analysis. J.W.G. also wrote the initial draft of the manuscript. L.A.S. is corresponding author, participated in experimental design, data analysis and critically edited the manuscript. Both authors read and approved the final manuscript.

## References

[B1] Tyler KL, Knipe DM and Howley PM (2001). Reoviruses. Fields Virology.

[B2] Golden JW, Linke J, Schmechel S, Thoemke K, Schiff LA (2002). Addition of exogenous protease facilitates reovirus infection in many restrictive cells. J Virol.

[B3] Golden JW, Bahe JA, Lucas WT, Nibert ML, Schiff LA (2004). Cathepsin S supports acid-independent infection by some reoviruses. J Biol Chem.

[B4] Lee PWK, Hayes EC, Joklik WK (1981). Protein sigma 1 is the reovirus cell attachment protein. Virology.

[B5] Weiner HL, Powers ML, Fields BN (1980). Absolute linkage of virulence and central nervous system cell tropism of reoviruses to viral hemagglutinin. Journal of Infectious Diseases.

[B6] Barton ES, Connolly JL, Forrest JC, Chappell JD, Dermody TS (2001). Utilization of sialic acid as a coreceptor enhances reovirus attachment by multistep adhesion strengthening. J Biol Chem.

[B7] Barton ES, Forrest JC, Connolly JL, Chappell JD, Liu Y, Schnell FJ, Nusrat A, Parkos CA, Dermody TS (2001). Junction adhesion molecule is a receptor for reovirus. Cell.

[B8] Helander A, Silvey KJ, Mantis NJ, Hutchings AB, Chandran K, Lucas WT, Nibert ML, Neutra MR (2003). The viral sigma1 protein and glycoconjugates containing alpha2-3-linked sialic acid are involved in type 1 reovirus adherence to M cell apical surfaces. J Virol.

[B9] Ehrlich M, Boll W, Van Oijen A, Hariharan R, Chandran K, Nibert ML, Kirchhausen T (2004). Endocytosis by random initiation and stabilization of clathrin-coated pits. Cell.

[B10] Sturzenbecker LJ, Nibert M, Furlong D, Fields BN (1987). Intracellular digestion of reovirus particles requires a low pH and is an essential step in the viral infectious cycle. J Virol.

[B11] Chang CT, Zweerink HJ (1971). Fate of parental reovirus in infected cell. Virology.

[B12] Borsa J, Sargent MD, Lievaart PA, Copps TP (1981). Reovirus: evidence for a second step in the intracellular uncoating and transcriptase activation process. Virology.

[B13] Silverstein SC, Astell C, Levin DH, Schonberg M, Acs G (1972). The mechanisms of reovirus uncoating and gene activation in vivo. Virology.

[B14] Nibert ML (1993). Structure and function of reovirus outer capsid proteins as they relate to early steps in infection. Microbiology and Molecular Genetics.

[B15] Jane-Valbuena J, Nibert ML, Spencer SM, Walker SB, Baker TS, Chen Y, Centonze VE, Schiff LA (1999). Reovirus virion-like particles obtained by recoating infectious subvirion particles with baculovirus-expressed sigma3 protein: an approach for analyzing sigma3 functions during virus entry. J Virol.

[B16] Chandran K, Farsetta DL, Nibert ML (2002). Strategy for nonenveloped virus entry: a hydrophobic conformer of the reovirus membrane penetration protein micro 1 mediates membrane disruption. J Virol.

[B17] Chandran K, Nibert ML (1998). Protease cleavage of reovirus capsid protein mu1/mu1C is blocked by alkyl sulfate detergents, yielding a new type of infectious subvirion particle. J Virol.

[B18] Chandran K, Walker SB, Chen Y, Contreras CM, Schiff LA, Baker TS, Nibert ML (1999). In vitro recoating of reovirus cores with baculovirus-expressed outer- capsid proteins mu1 and sigma3. J Virol.

[B19] Shepard DA, Ehnstrom JG, Schiff LA (1995). Association of reovirus outer capsid proteins sigma 3 and mu 1 causes a conformational change that renders sigma 3 protease sensitive. J Virol.

[B20] Jane-Valbuena J, Breun LA, Schiff LA, Nibert ML (2002). Sites and determinants of early cleavages in the proteolytic processing pathway of reovirus surface protein sigma3. J Virol.

[B21] Baer GS, Ebert DH, Chung CJ, Erickson AH, Dermody TS (1999). Mutant cells selected during persistent reovirus infection do not express mature cathepsin L and do not support reovirus disassembly. J Virol.

[B22] Baer GS, Dermody TS (1997). Mutations in reovirus outer-capsid protein sigma3 selected during persistent infections of L cells confer resistance to protease inhibitor E64. J Virol.

[B23] Ebert DH, Deussing J, Peters C, Dermody TS (2002). Cathepsin L and cathepsin B mediate reovirus disassembly in murine fibroblast cells. J Biol Chem.

[B24] Ebert DH, Wetzel JD, Brumbaugh DE, Chance SR, Stobie LE, Baer GS, Dermody TS (2001). Adaptation of reovirus to growth in the presence of protease inhibitor E64 segregates with a mutation in the carboxy terminus of viral outer-capsid protein sigma3. J Virol.

[B25] Kothandaraman S, Hebert MC, Raines RT, Nibert ML (1998). No role for pepstatin-A-sensitive acidic proteinases in reovirus infections of L or MDCK cells. Virology.

[B26] Martinez CG, Guinea R, Benavente J, Carrasco L (1996). The entry of reovirus into L cells is dependent on vacuolar proton-ATPase activity. J Virol.

[B27] Canning WM, Fields BN (1983). Ammonium chloride prevents lytic growth of reovirus and helps to establish persistent infection in mouse L cells. Science.

[B28] Wilson GJ, Nason EL, Hardy CS, Ebert DH, Wetzel JD, Venkataram Prasad BV, Dermody TS (2002). A single mutation in the carboxy terminus of reovirus outer-capsid protein sigma 3 confers enhanced kinetics of sigma 3 proteolysis, resistance to inhibitors of viral disassembly, and alterations in sigma 3 structure. J Virol.

[B29] Kirschke H, Wiederanders B, Bromme D, Rinne A (1989). Cathepsin S from bovine spleen. Purification, distribution, intracellular localization and action on proteins. Biochem J.

[B30] Chandran K, Sullivan NJ, Felbor U, Whelan SP, Cunningham JM (2005). Endosomal Proteolysis of the Ebola Virus Glycoprotein Is Necessary for Infection. Science.

[B31] Bodkin DK, Nibert ML, Fields BN (1989). Proteolytic digestion of reovirus in the intestinal lumens of neonatal mice. J Virol.

[B32] Bass DM, Bodkin D, Dambrauskas R, Trier JS, Fields BN, Wolf JL (1990). Intraluminal proteolytic activation plays an important role in replication of type 1 reovirus in the intestines of neonatal mice. J Virol.

[B33] Jackson GG, Muldoon RL, Cooper RS (1961). Reovirus type 1 as an etiologic agent of the common cold. J Clin Invest.

[B34] Morin MJ, Warner A, Fields BN (1994). A pathway for entry of reoviruses into the host through M cells of the respiratory tract. Journal of Experimental Medicine.

[B35] Morin MJ, Warner A, Fields BN (1996). Reovirus infection in rat lungs as a model to study the pathogenesis of viral pneumonia. J Virol.

[B36] Lee WL, Downey GP (2001). Leukocyte elastase: physiological functions and role in acute lung injury. Am J Respir Crit Care Med.

[B37] Kirschke H, Barrett AJ, Rawlings ND (1998). Lysosomal cysteine proteases.

[B38] Shapiro SD (2002). Neutrophil elastase: path clearer, pathogen killer, or just pathologic?. Am J Respir Cell Mol Biol.

[B39] Belaaouaj A (2002). Neutrophil elastase-mediated killing of bacteria: lessons from targeted mutagenesis. Microbes Infect.

[B40] Stockley RA (1999). Neutrophils and protease/antiprotease imbalance. Am J Respir Crit Care Med.

[B41] Churg A, Wright JL (2005). Proteases and emphysema. Curr Opin Pulm Med.

[B42] Yasui K, Baba A, Iwasaki Y, Kubo T, Aoyama K, Mori T, Yamazaki T, Kobayashi N, Ishiguro A (2005). Neutrophil-mediated inflammation in respiratory syncytial viral bronchiolitis. Pediatr Int.

[B43] Farone AL, Frevert CW, Farone MB, Morin MJ, Fields BN, Paulauskis JD, Kobzik L (1996). Serotype-dependent induction of pulmonary neutrophilia and inflammatory cytokine gene expression by reovirus. J Virol.

[B44] London L, Majeski EI, Paintlia MK, Harley RA, London SD (2002). Respiratory reovirus 1/L induction of diffuse alveolar damage: a model of acute respiratory distress syndrome. Exp Mol Pathol.

[B45] Senior RM, Campbell EJ, Landis JA, Cox FR, Kuhn C, Koren HS (1982). Elastase of U-937 monocytelike cells. Comparisons with elastases derived from human monocytes and neutrophils and murine macrophagelike cells. J Clin Invest.

[B46] Barrett AJ, Kembhavi AA, Brown MA, Kirschke H, Knight CG, Tamai M, Hanada K (1982). L-trans-Epoxysuccinyl-leucylamido(4-guanidino)butane (E-64) and its analogues as inhibitors of cysteine proteinases including cathepsins B, H and L. Biochem J.

[B47] Nathanson CM, Wasselius J, Wallin H, Abrahamson M (2002). Regulated expression and intracellular localization of cystatin F in human U937 cells. Eur J Biochem.

[B48] Bowman EJ, Siebers A, Altendorf K (1988). Bafilomycins: a class of inhibitors of membrane ATPases from microorganisms, animal cells, and plant cells. Proc Natl Acad Sci U S A.

[B49] Maxfield FR (1982). Weak bases and ionophores rapidly and reversibly raise the pH of endocytic vesicles in cultured mouse fibroblasts. J Cell Biol.

[B50] Ohkuma S, Poole B (1978). Fluorescence probe measurement of the intralysosomal pH in living cells and the perturbation of pH by various agents. Proc Natl Acad Sci U S A.

[B51] Welgus HG, Senior RM, Parks WC, Kahn AJ, Ley TJ, Shapiro SD, Campbell EJ (1992). Neutral proteinase expression by human mononuclear phagocytes: a prominent role of cellular differentiation. Matrix Suppl.

[B52] Picone R, Kajtaniak EL, Nielsen LS, Behrendt N, Mastronicola MR, Cubellis MV, Stoppelli MP, Pedersen S, Dano K, Blasi F (1989). Regulation of urokinase receptors in monocytelike U937 cells by phorbol ester phorbol myristate acetate. J Cell Biol.

[B53] Powers JC, Gupton BF, Harley AD, Nishino N, Whitley RJ (1977). Specificity of porcine pancreatic elastase, human leukocyte elastase and cathepsin G. Inhibition with peptide chloromethyl ketones. Biochim Biophys Acta.

[B54] Salvesen G, Enghild JJ (1990). An unusual specificity in the activation of neutrophil serine proteinase zymogens. Biochemistry.

[B55] Atkins KB, Troen BR (1995). Phorbol ester stimulated cathepsin L expression in U937 cells. Cell Growth Differ.

[B56] Hanson RD, Connolly NL, Burnett D, Campbell EJ, Senior RM, Ley TJ (1990). Developmental regulation of the human cathepsin G gene in myelomonocytic cells. J Biol Chem.

[B57] Yoshimura K, Crystal RG (1992). Transcriptional and posttranscriptional modulation of human neutrophil elastase gene expression. Blood.

[B58] Greco MN, Hawkins MJ, Powell ET, Almond HRJ, Corcoran TW, de Garavilla L, Kauffman JA, Recacha R, Chattopadhyay D, Andrade-Gordon P, Maryanoff BE (2002). Nonpeptide inhibitors of cathepsin G: optimization of a novel beta-ketophosphonic acid lead by structure-based drug design. J Am Chem Soc.

[B59] de Duve C (1975). Exploring cells with a centrifuge. Science.

[B60] Dahlgren C, Carlsson SR, Karlsson A, Lundqvist H, Sjolin C (1995). The lysosomal membrane glycoproteins Lamp-1 and Lamp-2 are present in mobilizable organelles, but are absent from the azurophil granules of human neutrophils. Biochem J.

[B61] Cham BP, Gerrard JM, Bainton DF (1994). Granulophysin is located in the membrane of azurophilic granules in human neutrophils and mobilizes to the plasma membrane following cell stimulation. Am J Pathol.

[B62] Saito N, Pulford KA, Breton-Gorius J, Masse JM, Mason DY, Cramer EM (1991). Ultrastructural localization of the CD68 macrophage-associated antigen in human blood neutrophils and monocytes. Am J Pathol.

[B63] Fittschen C, Henson PM (1994). Linkage of azurophil granule secretion in neutrophils to chloride ion transport and endosomal transcytosis. J Clin Invest.

[B64] Hallett MB (1989). The Neutrophil : cellular biochemistry and physiology.

[B65] Owen CA, Campbell MA, Boukedes SS, Campbell EJ (1997). Cytokines regulate membrane-bound leukocyte elastase on neutrophils: a novel mechanism for effector activity. Am J Physiol.

[B66] Juffrie M, van Der Meer GM, Hack CE, Haasnoot K, Veerman AJ, Thijs LG, Sutaryo (2000). Inflammatory mediators in dengue virus infection in children: interleukin-8 and its relationship to neutrophil degranulation. Infect Immun.

[B67] Jaovisidha P, Peeples ME, Brees AA, Carpenter LR, Moy JN (1999). Respiratory syncytial virus stimulates neutrophil degranulation and chemokine release. J Immunol.

[B68] Hamamdzic D, Altman-Hamamdzic S, Bellum SC, Phillips-Dorsett TJ, London SD, London L (1999). Prolonged induction of IL-8 gene expression in a human fibroblast cell line infected with reovirus serotype 1 strain Lang. Clin Immunol.

[B69] Furlong DB, Nibert ML, Fields BN (1988). Sigma 1 protein of mammalian reoviruses extends from the surfaces of viral particles. J Virol.

[B70] Silverstein SC, Dales S (1968). The penetration of reovirus RNA and initiation of its genetic function in L-strain fibroblasts. J Cell Biol.

[B71] Broering TJ, McCutcheon AM, Centonze VE, Nibert ML (2000). Reovirus nonstructural protein muNS binds to core particles but does not inhibit their transcription and capping activities. J Virol.

